# Supplementary material for: Characterizing the landscape of gene expression variance in humans
Source: PLoS Genet. 2023 Jul 6;19(7):e1010833. doi: 10.1371/journal.pgen.1010833 (PMC10353820; doi:10.1371/journal.pgen.1010833)
Supplement: S1 Fig — (PDF) [file pgen.1010833.s001.pdf]

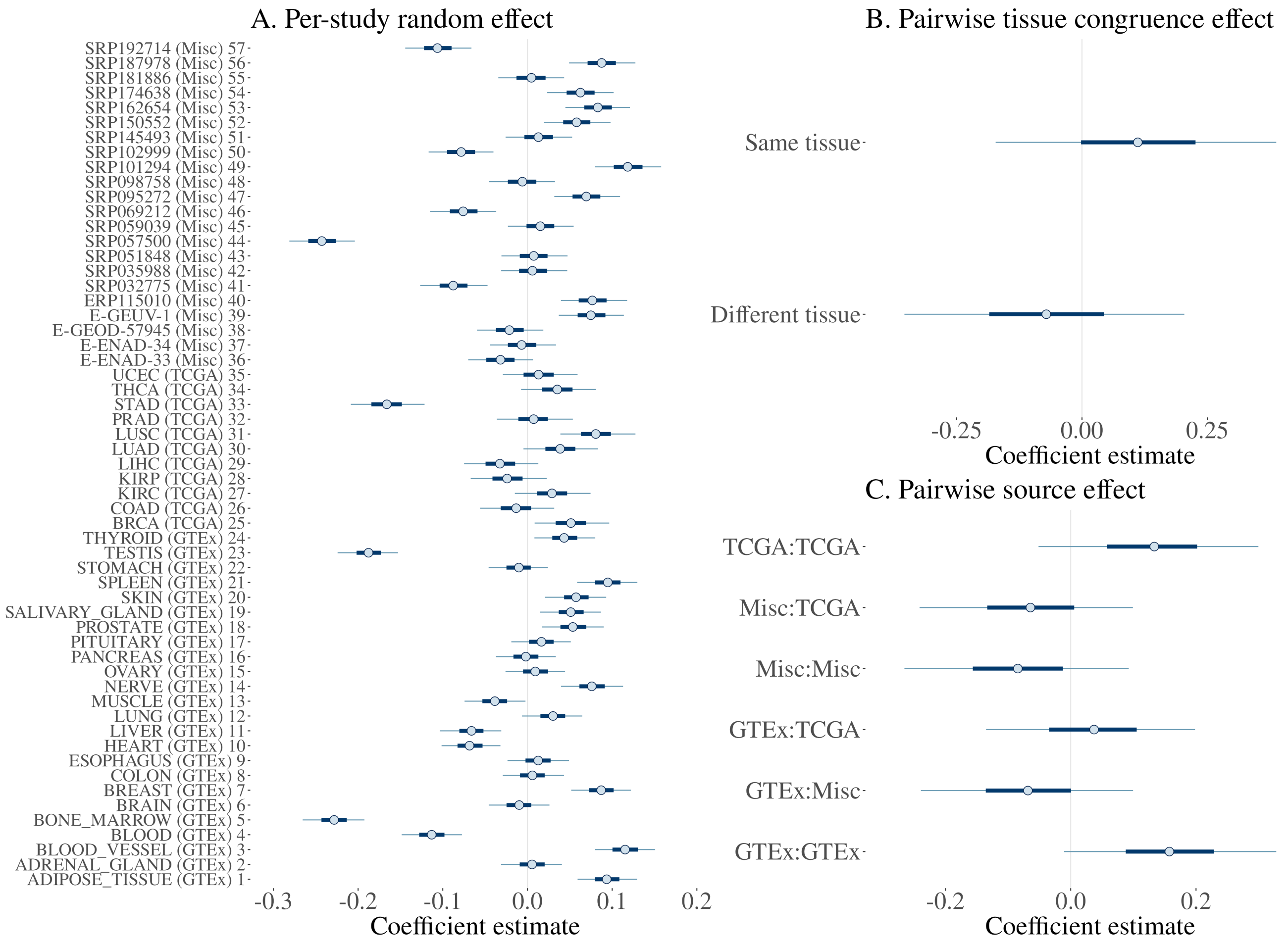

S1 Fig: Modeling the correlations between transcriptional variance across studies. The panels show coefficient estimates from a linear model using the among studies Spearman correlations between gene expression SDs as the response variable. These correlations are shown in fig. 2, panels A and B. In the linear model (see Methods for model equation), correlations are Fisher z-transformed. Study source and tissue are added as fixed effects. Coefficient estimates are shown with 50% and 95% credibility intervals. (A) The per-study random effect which accounts for the non-independence between the pairwise correlation values and estimates the characteristic contribution of each study to these correlations. For example, the lowest estimate among these parameters, which corresponds to the study BONE MARROW (from GTEx), indicates that correlations involving this study tend to be lower than the others. (B) and (C) Fixed effect estimates for the effects of tissue congruence and study-source effect. In (B) we see that correlations among studies that use the same tissue are slightly higher; and (C) correlations involving studies in the "Misc." category (non-GTEx and non-TCGA) tend to be lower, while comparisons involving GTEx and TCGA are higher.
