# Supplementary material for: Characterizing the landscape of gene expression variance in humans
Source: PLoS Genet. 2023 Jul 6;19(7):e1010833. doi: 10.1371/journal.pgen.1010833 (PMC10353820; doi:10.1371/journal.pgen.1010833)
Supplement: S2 Fig — (PDF) [file pgen.1010833.s002.pdf]

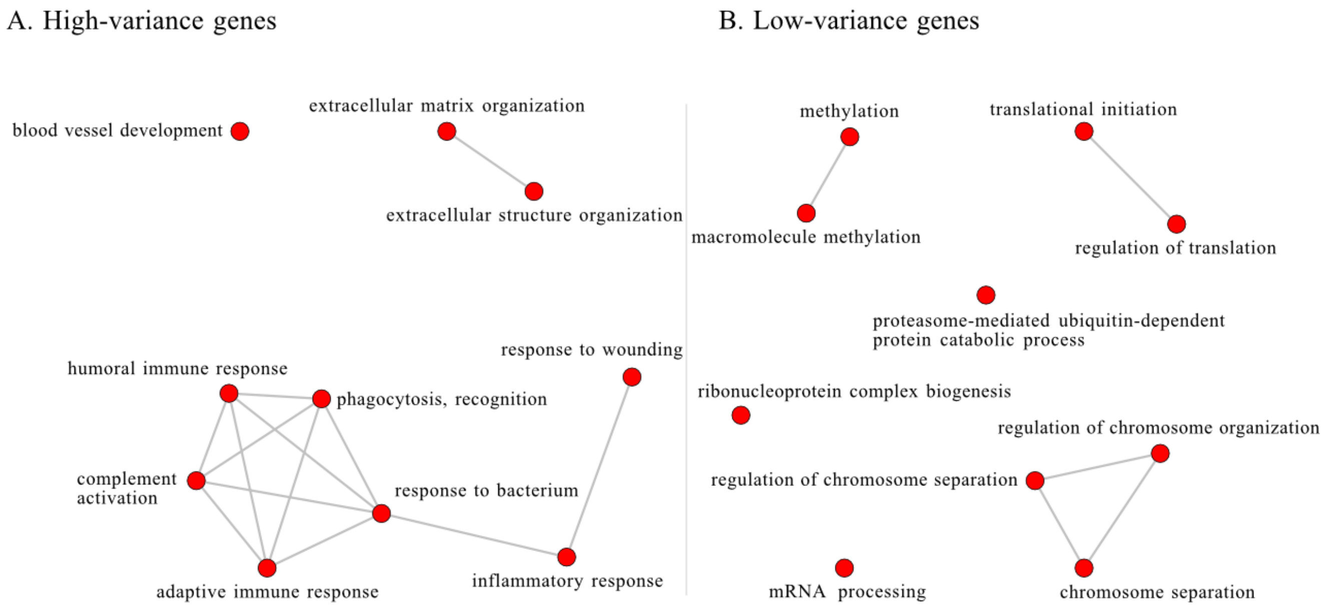

S2 Fig: Gene set enrichment analyses testing for over-representation of gene ontology categories in the upper and lower 5% quantiles of the gene variance rank. (A) High-variance genes are enriched for terms related to immune function, response to wounding, blood vessel morphogenesis, and inflammatory response. In contrast, (B) low-variance genes are associated with translation, control of methylation, RNA processing, chromosome separation, and other cell housekeeping functions. All displayed terms are significant with a 5% FDR corrected p-value below 10^{-3}.
