# Supplementary material for: Characterizing the landscape of gene expression variance in humans
Source: PLoS Genet. 2023 Jul 6;19(7):e1010833. doi: 10.1371/journal.pgen.1010833 (PMC10353820; doi:10.1371/journal.pgen.1010833)
Supplement: S3 Fig — (PDF) [file pgen.1010833.s003.pdf]

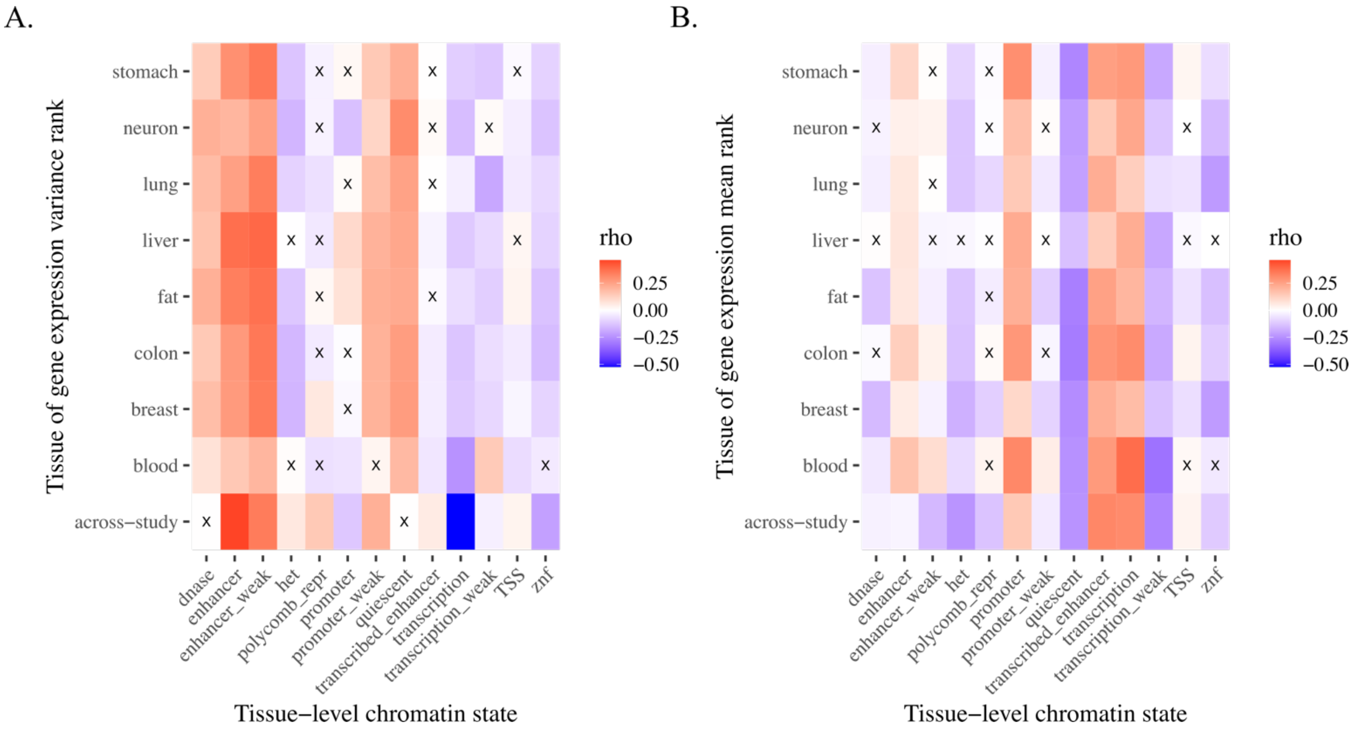

S3 Fig: Across-study and tissue-specific gene expression variance and mean correlations with non-overlapping chromatin states through ChromHMM[1]. The across-study variance (A) and mean (B) rank metrics ("across-study" on y-axis) were associated with universal chromatin states[2] (x-axis). The tissue-level variance (A) and mean (B) rank metrics (see Methods; Supplementary Table 1; named tissues on y-axis) were associated with their respective tissue-specific chromatin states[3] (x-axis, see Supplementary Table 1). Boxes marked with an "X" are not significantly correlated; all other comparisons are significant (Benjamini-Hochberg adjusted $p < 0.05$). Het indicates heterochromatin; TSS, transcription start sites; znf, zinc finger genes.

References

1. Ernst, J. & Kellis, M. ChromHMM: Automating chromatin-state discovery and characterization. *Nature Methods* vol. 9 (2012).
2. Vu, H. & Ernst, J. Universal annotation of the human genome through integration of over a thousand epigenomic datasets. *Genome Biol.* **23**, 9 (2022).
3. Ernst, J. & Kellis, M. Large-scale imputation of epigenomic datasets for systematic annotation of diverse human tissues. *Nat. Biotechnol.* **33**, 364–76 (2015).
