# Supplementary material for: Characterizing the landscape of gene expression variance in humans
Source: PLoS Genet. 2023 Jul 6;19(7):e1010833. doi: 10.1371/journal.pgen.1010833 (PMC10353820; doi:10.1371/journal.pgen.1010833)
Supplement: S4 Fig — (PDF) [file pgen.1010833.s004.pdf]

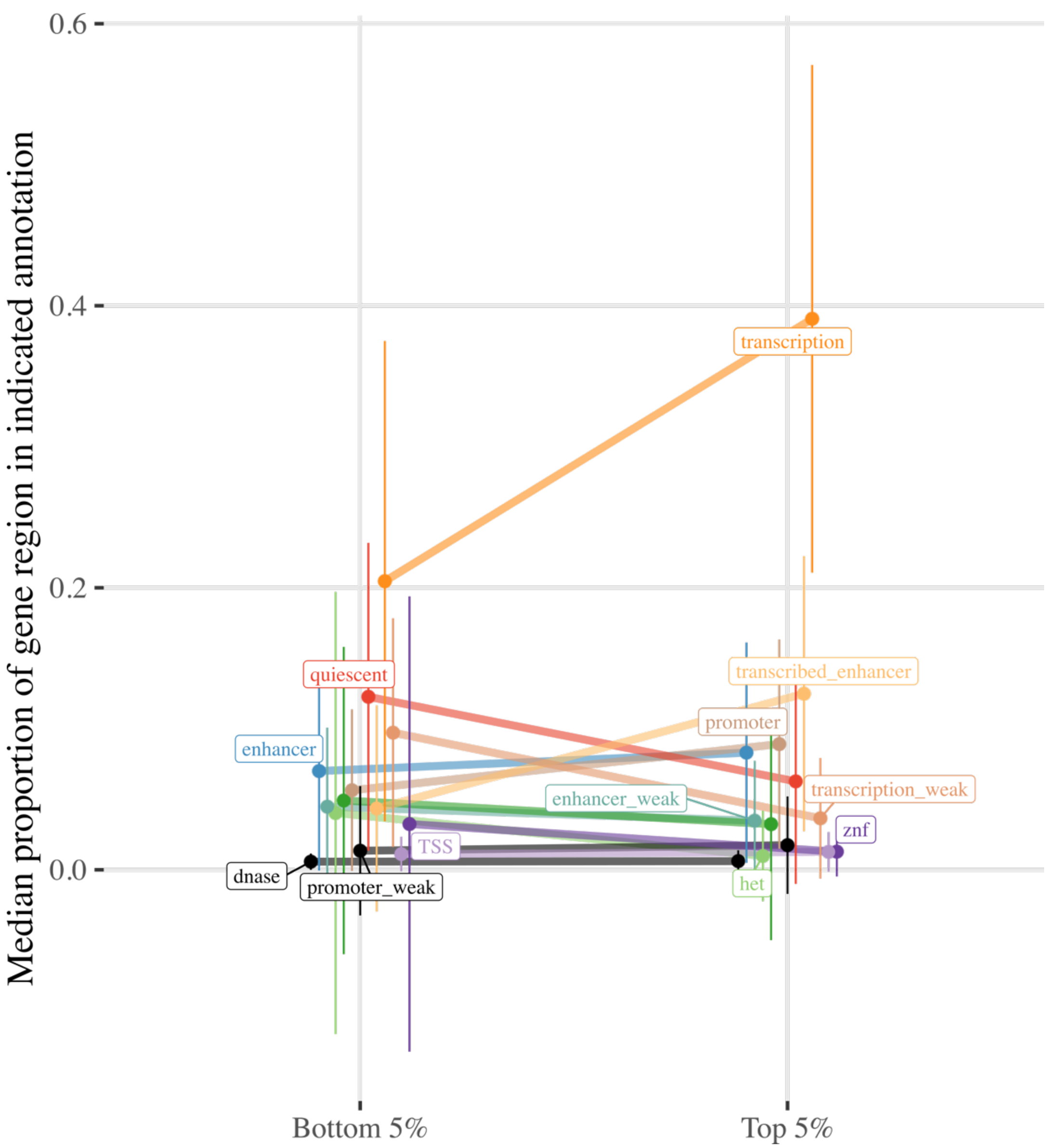

S4 Fig: Proportion of gene regions made up of ChromHMM chromatin states for genes in the top and bottom 5% of the across-study mean rank metric. Line plot contrasts the proportion of gene regions made up of the indicated chromatin states for genes in the top and bottom 5% of the across-study mean rank metric. Ends denote the median proportion of gene regions made up of the chromatin state, and error bars represent the standard error of the mean (SEM). States colored black are not significant, all others exhibit significant differences in gene region made up of the chromatin state for genes in the top and bottom 5% of the mean rank metric (Benjamini-Hochberg adjusted $p_{Wilcoxon} < 0.05$). Het indicates heterochromatin; TSS, transcription start sites; znf, zinc finger genes.
