# Supplementary material for: Characterizing the landscape of gene expression variance in humans
Source: PLoS Genet. 2023 Jul 6;19(7):e1010833. doi: 10.1371/journal.pgen.1010833 (PMC10353820; doi:10.1371/journal.pgen.1010833)
Supplement: S5 Fig — (PDF) [file pgen.1010833.s005.pdf]

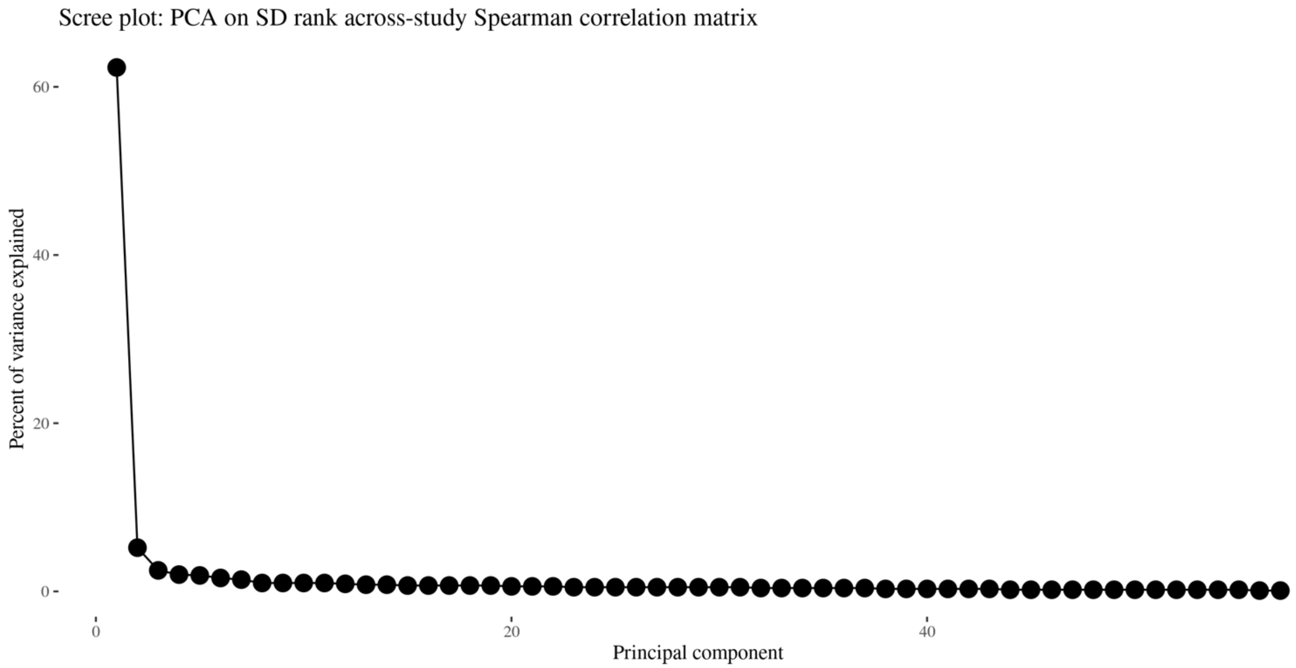

S5 Fig: Scree plot on SD rank across-study Spearman correlation matrix. Scree plot shows the percent of variance explained by each PC. These PCs are calculated on the across-study Spearman correlation matrix of across-study SD expression rank. PC1 accounts for 62.3% of variation and PC2, 5.2%.
