# Supplementary material for: Characterizing the landscape of gene expression variance in humans
Source: PLoS Genet. 2023 Jul 6;19(7):e1010833. doi: 10.1371/journal.pgen.1010833 (PMC10353820; doi:10.1371/journal.pgen.1010833)
Supplement: S6 Fig — (PDF) [file pgen.1010833.s006.pdf]

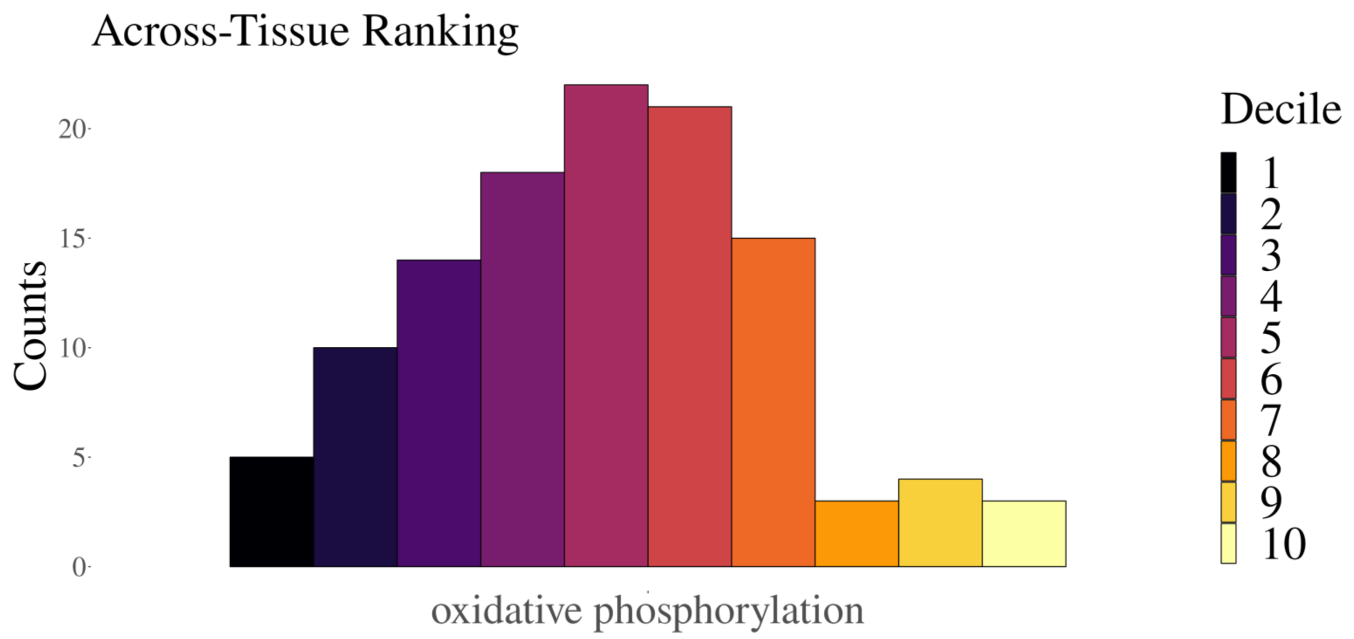

S6 Fig: Distributions of decile ranks of the GO term oxidative phosphorylation. The plot shows the count of genes in each decile of the SD rank. Oxidative phosphorylation is the only outlier that shows both low Shannon entropy and low skewness. This term being the only outlier in the skewness-by-variance relation is interesting, as cell respiration has a particular mixture of being both a base cell process [1], like the other low-variance biased terms, and being a process that must react to the environmental conditions in a dynamic fashion [2, 3], similar to the other high-variance biased terms.

While we cannot be certain of what is driving the enrichment for intermediate-variance genes, this combination is suggestive that the enrichment for intermediate variance genes is related to a combination of the selective regimes we outline above.

References

1. Das J. The role of mitochondrial respiration in physiological and evolutionary adaptation. Bioessays. 2006 Sep;28(9):890–901.

2. Caja S, Enrıíquez JA. Mitochondria in endothelial cells: Sensors and integrators of environmental cues. Redox Biol. 2017 Aug;12:821–7.

3. Hoppins S. The regulation of mitochondrial dynamics. Curr Opin Cell Biol. 2014 Aug;29:46–52.
