# Supplementary material for: Characterizing the landscape of gene expression variance in humans
Source: PLoS Genet. 2023 Jul 6;19(7):e1010833. doi: 10.1371/journal.pgen.1010833 (PMC10353820; doi:10.1371/journal.pgen.1010833)
Supplement: S1 Table — (PDF) [file pgen.1010833.s007.pdf]

S1 Table: Variance and mean rank metrics and the corresponding ChromHMM annotations used.

Variance/mean rank metric	ChromHMM annotation	Roadmap ID
Across-study	Universal[2]	
Blood	Primary mononuclear cells from peripheral blood	E062
Breast	Breast Myoepithelial Primary Cells	E027
Colon	Sigmoid Colon	E106
Fat	Adipose Nuclei	E063
Liver	Liver	E066
Lung	Lung	E096
Neuron	H9 Derived Neuron Cultured Cells	E010
Stomach	Stomach Smooth Muscle	E111
