# Supplementary material for: Characterizing the landscape of gene expression variance in humans
Source: PLoS Genet. 2023 Jul 6;19(7):e1010833. doi: 10.1371/journal.pgen.1010833 (PMC10353820; doi:10.1371/journal.pgen.1010833)
Supplement: Attachment — Submitted filename: peerreview_plosgenet20230128.docx [file pgen.1010833.s016.docx]

**Summary**: This paper examines the landscape of variance in gene expression using various public bulk RNA-seq data. It ranks genes according to their variance and systematically examines the association between high or low variance genes and their function and epigenetic signature.

The study provides important information on gene expression variance. The study is well designed and the manuscript is well written. On the other hand, the interpretation of the results is sometimes inadequate or misleading. I recommend the publications of this manuscript after the revision.

**Major comment**

The relevant part of the comment in the text is written in italics.

**Comment1:**

**What is the difference between the analysis using Skeveness and entoropy shown in Fig. 3 and the Gene Ontology Enrichment analysis? Both appear to search for gene sets associated with high or low variance gene sets. For why did you perform these two different analyses?**

**Comment2:**

*(Result) However, all these pairwise effects are mild, and the largest effects on the correlations are those associated with individual studies, in particular some specific tissues, i.e., comparisons involving BONE MARROW (from GTEx) and study SRP057500 (which used*

*platelets) are on average lower (SI fig. 1 A).*

**Recent studies have shown that cellular subset heterogeneity affects the variance of bulk gene expression (**[**https://www.sciencedirect.com/science/article/pii/S2001037022003968**](https://www.sciencedirect.com/science/article/pii/S2001037022003968)**). For example, when high and low gene expressing subsets are mixed in a sample, the dispersion of the cellular subset fraction between samples affects the gene expression variance.**

**Considering the diversity of immune cell subsets and their individual differences, It seems this result is reasonable and not surprising. How can these results be interpreted in terms of cellular subset heterogeneity?**

Comment3:

*> (Discussion) We find a broadly similar pattern of transcriptional variance, evidenced by the high correlations between gene expression variance acrossmost studies, consistent withmeasurements of expression variance in single cells and in populations of cells for various tissues [6,16,29].*

**I think this sentence is misleading. expression variance in bulk samples and "cell-to-cell variability" quantified in single cell studies are different concept (See the second paragraph in Discussion Section in (**[**https://www.sciencedirect.com/science/article/pii/S2001037022003968**](https://www.sciencedirect.com/science/article/pii/S2001037022003968)**). This is because in a bulk experiment, the "cell-to-cell variability" information is averaged out. It is not surprising that they share a common cause, but this difference should be clarified and discussed to prevent readers from misunderstanding.**

**Minor comment**

**Comment1: In Fig2(D), It is unclear what the many coloured lines represent; please specify in Legend.**
