# Supplementary material for: Characterizing the landscape of gene expression variance in humans
Source: PLoS Genet. 2023 Jul 6;19(7):e1010833. doi: 10.1371/journal.pgen.1010833 (PMC10353820; doi:10.1371/journal.pgen.1010833)
Supplement: Attachment — Submitted filename: peerreview_plosgenet20230128_JFA_FINAL.docx [file pgen.1010833.s017.docx]

### Dear Dr. Cai,

### Thank you for forwarding the reviews for our recent submission to *PLoS Genetics*, “Characterizing the landscape of gene expression variance in humans” (PGENETICS-D-23-00013). We were pleased to read that the reviewers viewed our work as timely, insightful, and of broad interest. The reviewers’ comments were highly constructive.

### We have now thoroughly revised the manuscript in response to their suggestions. Major changes in this revision include:

### (i) A more thorough discussion on the levels of gene expression variation we are exploring to avoid confusion over interpretation, as pointed out by reviewer #1.

### (ii) A clearer description of the statistical models used in the manuscript.

### (iii) A better methodological choice suggested by reviewer #2.

### Our responses to the complete set of reviewer concerns are provided in detail below, with our point-by-point responses provided in blue indented text after each of the original comments.

### Please let us know if you need any further information.

### Sincerely,

### Julien Ayroles (on behalf of all authors)

**Reviewer #1:**

**Summary**: This paper examines the landscape of variance in gene expression using various public bulk RNA-seq data. It ranks genes according to their variance and systematically examines the association between high or low variance genes and their function and epigenetic signature.

The study provides important information on gene expression variance. The study is well designed and the manuscript is well written. On the other hand, the interpretation of the results is sometimes inadequate or misleading. I recommend the publications of this manuscript after the revision.

**Major comment**

The relevant part of the comment in the text is written in italics.

**Comment 1:**

What is the difference between the analysis using Skeveness and entoropy shown in Fig. 3 and the Gene Ontology Enrichment analysis? Both appear to search for gene sets associated with high or low variance gene sets. For why did you perform these two different analyses

### The Gene Ontology enrichment analysis (SI figure 2) looks for GO terms enriched in the top 5% and bottom 5% of the variance ranking. This means that only a fraction of the genes are used in this analysis. Meanwhile, the skewness by entropy analysis, shown in Figures 3 and 4, goes from GO terms to genes. It uses all ranked genes to investigate the relationship between variance and GO annotations.

### We believe these analyses to be complementary, and they allow us to investigate different aspects of the relationship between variance and function, i.e., the biological process terms associated with the extremes of the variance rank and the pattern of variance within each Gene Ontology term. We now note this on lines 157-159.

**Comment 2:**

*(Result) However, all these pairwise effects are mild, and the largest effects on the correlations are those associated with individual studies, in particular some specific tissues, i.e., comparisons involving BONE MARROW (from GTEx) and study SRP057500 (which used*

*platelets) are on average lower (SI fig. 1 A).*

**Recent studies have shown that cellular subset heterogeneity affects the variance of bulk gene expression (**[**https://www.sciencedirect.com/science/article/pii/S2001037022003968**](https://www.sciencedirect.com/science/article/pii/S2001037022003968)**). For example, when high and low gene expressing subsets are mixed in a sample, the dispersion of the cellular subset fraction between samples affects the gene expression variance.**

**Considering the diversity of immune cell subsets and their individual differences, It seems this result is reasonable and not surprising. How can these results be interpreted in terms of cellular subset heterogeneity?**

### This is an interesting point that likely applies to all studies using bulk measurements; those with more heterogeneous cell proportion or types are likely to be more susceptible to cell composition variability across different samples. How this relates to what we observe in the more divergent tissues (highlighted in the passage) is not clear. One possibility is that bone marrow is a particularly heterogeneous tissue from which relatively small samples are taken, which could lead to high between-sample variation and the low correlations with other tissues. As for platelets, given that only a single cell type is used, cell heterogeneity likely is not a factor, and the driving mechanisms of the divergent pattern of variation really is a difference in the gene expression variance in that tissue. One key observation to highlight is that many of the samples are from blood and white blood cells, and these are highly similar (contributing to the higher correlation we detect across samples originating from the same tissue). So, at least for circulating white blood cells, cell type heterogeneity does not seem to be a significant factor driving differences across studies. In any event, we now mention cell proportion heterogeneity as a source of variation in the discussion (lines 313-314).

**Comment 3:**

*> (Discussion) We find a broadly similar pattern of transcriptional variance, evidenced by the high correlations between gene expression variance across most studies, consistent with measurements of expression variance in single cells and in populations of cells for various tissues [6,16,29].*

**I think this sentence is misleading. expression variance in bulk samples and "cell-to-cell variability" quantified in single cell studies are different concept (See the second paragraph in Discussion Section in (**[**https://www.sciencedirect.com/science/article/pii/S2001037022003968**](https://www.sciencedirect.com/science/article/pii/S2001037022003968)**). This is because in a bulk experiment, the "cell-to-cell variability" information is averaged out. It is not surprising that they share a common cause, but this difference should be clarified and discussed to prevent readers from misunderstanding.**

### This section refers to the results from Dong *et al* 2011 suggesting that within cell variability is predictive of the variation across a population of cells. We have updated this paragraph to avoid any confusion between single cell and bulk expression variance. Also, see the added paragraph in the results in response to comments from reviewer #2 (lines 65-82) that clearly distinguishes the different levels of variation that are possible in gene expression data.

### ***Reference****: Dong, D., Shao, X., Deng, N., & Zhang, Z. (2011). Gene expression variations are predictive for stochastic noise. Nucleic acids research, 39(2), 403-413.* doi.org/10.1093/nar/gkq844,

**Minor comment:**

**Comment 1:** In Fig2(D), It is unclear what the many coloured lines represent; please specify in Legend.

### We updated the legend to read as follows:

### (D) Density plot of standard deviations, grouped and colored by study, after z-normalization. The inset plot shows the distribution of mean-centered standard deviations, grouped and colored by study, without normalization.

**Reviewer #2:**

**Summary:** The authors examine the variance of gene expression in 4200 expressed genes collected in 57 studies, including GTEx and TCGA. "Study" here refers to study-tissue combination, so for example GTEx contributes multiple studies (one per tissue) all deriving from the same "study origin". For each study, gene expression variance is calculated for each gene in a way that decorrelates from that gene's overall mean expression. The gene variances are ranked within study, and those ranks are used to compare gene variances across studies, so as to identify genes that are consistently high variance or low variance regardless of study. These results are then used to identify trends gene variance across tissues and in relation to gene function, and this provides the basis for discussion about evolutionary aspects of variance with regard to stabilizing selection vs dynamic biological function. In particular, lower gene variance is seen for house keeping genes whereas higher variance is seen for genes involved in responsive processes.

The methods and analyses used are appropriate, mostly clear, and in many cases innovative. The arguments made by the authors seem mostly well-supported by the data, and the manuscript is for the most part well written and persuasive.

### Thank you for the kind comments.

I have some concerns about the message of the manuscript and the descriptions of the methods, and a number of minor comments.

**1.** The Methods section "Correlations in transcriptional variance" needs more detail and explanation. Lines 398-410 describe a Bayesian

"varying effects" model, by which the authors presumably mean a Bayesian "random effects" or "mixed" model (and if so then should amend this), to model the Fisher transform of the correlations rho_ij. The authors do not define i and j but need to. Indeed, it would benefit this section to use notation earlier, eg, define the G x C matrix D containing elements s_gc, the normalized standard deviation of gene g=1,...,G in study i=1,...,C, and so on. The model specification needs more detail and some clarifications. The variance parameter of the normal description is written as sigma; better to stick with convention and call this sigma^2. It is not stated whether you fit the model to rho_ij for {i,j: i > j} or all i and j, or what. The design matrix X measures "the effects of tissue congruence and study-origin congruence" but the predictors should be stated clearly, eg, two indicator variables, one for same tissue and the other for same study-origin, where "study origin" is also defined explicitly. The priors could also be defined more precisely: is the alpha prior centered at zero? Is a unit normal prior what most people call a standard normal N(0,1), and unit exponential the same as Exp(1)?

### This is indeed a good point. We now describe in more detail the calculation of the across-study SD Spearman correlation matrix in the methods section (lines 426-432). We have also reformulated the model specification to match the implemented model exactly (see lines 433-451). Now, all the priors and coefficients are explicitly defined in the mathematical expression of the model.

**2.** There has recently been increasing interest in genetic and non-genetic effects on phenotypic variance that are separate from effects on phenotypic means (eg, PMID:20585554, PMID:21467569, PMID: 23150607, PMID:33326753, PMID:30389794), often called phenotypic variability or variance heterogeneity, but also variance. In light of those studies, it is important to emphasize that this study is not about gene expression variability. For example, within a population with a mean-controlling eQTL at high MAF, the gene expression variance could in theory entirely be due to that eQTL. Similarly, the reason why genes have similar expression variance ranks between studies from the same study origin (eg, between different tissue studies in GTEx) could entirely be due to the fact they were collected on the same genetic population, whose gene expression variance could be determined by the same eQTL effects on the expression means. Therefore, when the authors describe gene expression variance as being separate from effects of the mean, it will be good to emphasize (more than once) that the variance being studied includes variance due to segregating eQTLs. It might even be useful to have a table describing the sources of gene expression variance and their explanation -- eQTLs, temporal fluctuations, environment effects, etc.

### Indeed, some samples are shared across the GTEx studies, but the similarities across the other studies give confidence that this is not the main driving force of our results. Also, not all GTEx samples are from the same individuals, which further suggests we are uncovering common properties of genes rather than idiosyncratic properties of GTEx individuals. We now comment on this in the discussion (see lines 307-309).

### We have also added a paragraph (lines 65-82) describing the different terminology that can be used to describe gene expression variance. We hope this new text makes it clear what level of variation we are working with.

**3.** Figure C upper panel was confusing me initially. Underneath the horizontal axis it says "Increasing Variance Rank" but the variances in context 2 are not monotonically increasing. Please clarify this figure.

### We were trying to point out that the increase in variance rank does not need to be due to a monotonic increase in variance. Genes can have intermediate rank because they have intermediate expression variance or because their gene expression variance is not consistent across conditions. The higher the correlations across studies, the more the rank will reflect consistently monotonic increases in variance. We now make a note of this in the figure legend in an effort to have the figure be easily interpretable.

**4.** Line 108: it would be useful to report the correlation between the mean ranks and the std ranks, which is notable in that it is neglible.

### Mean-sd rank Spearman correlation is -0.07 and was added to this section (lines 220-221). This suggests that there is indeed some weak residual effect of mean expression on variance, probably driven by low-count genes, given the negative correlation. While significant, this effect is quite small.

**5.** Line 434-438. In the calculation of gene connectivity, the authors trim insignificant edges to each gene, then calculate the average correlation weight of the remaining edges. Does this mean that a gene with ten significant edges of average weight 0.4 will score as less connected than a gene with two significant edges of weight 0.5?

### Yes, this is correct. This comment made us revisit this choice and instead use the weighted sum of the edges (not the weighted mean). This should be a better representation of the level of constraint of a given gene. We still take the average across studies. This change is now described at lines 475-480 and did not affect the conclusions (lines 224-226).

**6.** Line 148 / Figure 3: What is the GO term at around (2.05, 0.08)? This one sticks out by having both low entropy and low skewness, suggesting a U-shaped distribution of std dev deciles. It would be good to include the data points for this plot in SI data 5 (I couldn't find it).

### The term at (2.10, 0.194) is “oxidative phosphorylation,” and indeed it has a bias towards intermediate variance, as shown in the plot below. This term being the only outlier in the skewness-entropy relation is interesting given its dual nature. This term is part of a base cell process, like the other low-variance biased terms, but his process must also react to environmental conditions in a dynamic fashion, similar to the other high-variance biased terms. While we can't be certain of what is driving the enrichment for intermediate-variance genes, this particular combination suggests that the enrichment for intermediate variance genes is related to a combination of the selective regimes we outline above. We have added this comment to the discussion (lines 343-346) and to the legend of an additional figure for the variance decile counts of this outlier term (SI figure 6). We now also provide a table with the skewness and entropy by level-3 GO term (SI data 6).

**7.** Line 376-377: "[DESeq2's vst] mean-variance correction was verified by plotting mean-variance relations before and after correction [plots in SI]". The authors use DESeq2's vst() function to remove the mean-variance relationship. That function applies an estimated variance stabilizing function that is generally fine but, because of its log-based construction, can work poorly for genes with low mean read counts, as seen for blood, colon, and stad. The authors should point this out and--assuming this is the case--reassure the reader overall conclusions are not affected.

### We have added a comment related to the studies which still have a residual mean-variance relation (see lines 408-410). In summary, given that these studies don't show up as outliers in any of our analyses, we don't expect this residual mean-variance relationship to be biasing the overall results or affecting our main conclusions.
