# Supplementary material for: Characterizing the landscape of gene expression variance in humans
Source: PLoS Genet. 2023 Jul 6;19(7):e1010833. doi: 10.1371/journal.pgen.1010833 (PMC10353820; doi:10.1371/journal.pgen.1010833)
Supplement: Attachment — Submitted filename: exp var response to reviewer v2.docx [file pgen.1010833.s018.docx]

Major comment:

The items listed in the manuscript's Supporting Information are not easily cross-referenced with the files in the corresponding github repository. I strongly suggest that, in addition to the github, the authors put the 15 SI items in some other kind of permanent storage (eg, Figshare, Dryad, etc) so readers can easily locate them.

### Thank you for the comment. We have added a README file in the SI folder on the github repository to facilitate the cross-reference between SI items and files in the repo. We have also deposited the SI files on Zenodo under doi: 10.5281/zenodo.8028690

Minor comment:

Line 446. The normal priors with variance 1/4 are described as weakly informative. Perhaps this counts as weak information, but without knowing the variance of rho_ij, 1/4 seems like it might impose a lot of shrinkage. Perhaps in parentheses, the authors could state the variance (or sd) of the rho_ij values, just to put the 1/4 (sd=1/2) in context.

### We have added (lines 448-449) information on what coefficient values are plausible under these priors (-1 to 1) and what the range of values is in the response variables (0 to 1.5). Given these compatible scales, we feel that the choice of priors for the fixed effects is justified, as we don't expect group mean differences to be larger than 1. Note that these parameters do not relate to the variance in the rho_ij values, but the differences between groups of comparisons. The residual standard deviation of the rho_ij values receives an exp(1) prior, which allows for much larger variances.
